# 

**Published:** 1861-07

**Authors:** 


Published Monthly, at $3 per Annum in Advance.

### ATLANTA MEDICAL AND SURGICAL JOURNAL.

J. G. WESTMORELAND, M. D.,
PROFESSOR OF MATERIA MEDICA AND MEDICAL JURISPRUDENCE IN THE ATLANTA MEDICAL COLLEGE,
EDITOR AND PROPRIETOR.

*Pax et scientia, sed veritas sine timore.*

VOL. VI. JULY, 1861. No. XI.

ATLANTA, GA:

ATLANTA INTELLIGENCER PRINT.

1861.

Each Number contains Sixty-Four Pages.

